# Regulatory T cells from patients with end-stage organ disease can be isolated, expanded and cryopreserved according good manufacturing practice improving their function

**DOI:** 10.1186/s12967-019-2004-2

**Published:** 2019-08-05

**Authors:** Francesca Ulbar, Tiziana Montemurro, Tatiana Jofra, Miriam Capri, Giorgia Comai, Valentina Bertuzzo, Cristiana Lavazza, Alessandra Mandelli, Mariele Viganò, Silvia Budelli, Maria Giulia Bacalini, Chiara Pirazzini, Paolo Garagnani, Valeria Giudice, Daria Sollazzo, Antonio Curti, Mario Arpinati, Gaetano La Manna, Matteo Cescon, Antonio Daniele Pinna, Claudio Franceschi, Manuela Battaglia, Rosaria Giordano, Lucia Catani, Roberto Massimo Lemoli

**Affiliations:** 1 Department of Experimental, Diagnostic and Specialty Medicine, Institute of Hematology “L. e A. Seràgnoli”, University of Bologna, Bologna, Italy; 2 Cell Factory, Unit of Cellular Therapy and Cryobiology, Fondazione IRCCS Ca’ Granda Ospedale Maggiore Policlinico, Milan, Italy; 3 Diabetes Research Institute, IRCCS San Raffaele Scientific Institute, Milan, Italy; 4 Department of Experimental, Diagnostic and Specialty Medicine, University of Bologna, Bologna, Italy; 5 Nephrology Dialysis and Renal Transplant Unit, Department of Experimental Diagnostic and Specialty Medicine, University of Bologna, Bologna, Italy; 6 Department of Medical and Surgical Sciences, University of Bologna, Bologna, Italy; 7 EPIGET LAB, Department of Clinical Sciences and Community Health, University of Milan, Milan, Italy; 8 IRCCS Istituto delle Scienze Neurologiche di Bologna, Bologna, Italy; 9 Clinical Chemistry, Department of Laboratory Medicine, Karolinska Institutet at Huddinge University Hospital, Stockholm, Sweden; 10 Laboratory of Cell Biology, Rizzoli Orthopaedic Institute, Bologna, Italy; 11 Unit of Bologna, CNR Institute of Molecular Genetics, Bologna, Italy; 12 Center for Applied Biomedical Research (CRBA), St. Orsola-Malpighi University Hospital, Bologna, Italy; 13 Azienda Ospedaliero-Universitaria di Bologna S. Orsola-Malpighi, Bologna, Italy; 14 Clinic of Hematology, Department of Internal Medicine (DiMI), University of Genoa, Genoa, Italy; 15 IRCCS Ospedale Policlinico S. Martino, Genoa, Italy

**Keywords:** End stage organ disease, Regulatory T cells, Safety, Murine model

## Abstract

**Background:**

Here, we isolated, expanded and functionally characterized regulatory T cells (Tregs) from patients with end stage kidney and liver disease, waiting for kidney/liver transplantation (KT/LT), with the aim to establish a suitable method to obtain large numbers of immunomodulatory cells for adoptive immunotherapy post-transplantation.

**Methods:**

We first established a preclinical protocol for expansion/isolation of Tregs from peripheral blood of LT/KT patients. We then scaled up and optimized such protocol according to good manufacturing practice (GMP) to obtain high numbers of purified Tregs which were phenotypically and functionally characterized in vitro and in vivo in a xenogeneic acute graft-versus-host disease (aGVHD) mouse model. Specifically, immunodepressed mice (NOD-SCID-gamma KO mice) received human effector T cells with or without GMP-produced Tregs to prevent the onset of xenogeneic GVHD.

**Results:**

Our small scale Treg isolation/expansion protocol generated functional Tregs. Interestingly, cryopreservation/thawing did not impair phenotype/function and DNA methylation pattern of *FOXP3* gene of the expanded Tregs. Fully functional Tregs were also isolated/expanded from KT and LT patients according to GMP. In the mouse model, GMP Tregs from LT or KT patient proved to be safe and show a trend toward reduced lethality of acute GVHD.

**Conclusions:**

These data demonstrate that expanded/thawed GMP-Tregs from patients with end-stage organ disease are fully functional in vitro. Moreover, their infusion is safe and results in a trend toward reduced lethality of acute GVHD in vivo, further supporting Tregs-based adoptive immunotherapy in solid organ transplantation.

**Electronic supplementary material:**

The online version of this article (10.1186/s12967-019-2004-2) contains supplementary material, which is available to authorized users.

## Background

Organ transplantation is the only curative treatment for patients affected by end-stage liver or kidney disease. Although immunosuppressive (IS) drugs have reduced the incidence of acute rejection and transplantation-associated mortality, their administration is associated with major side effects, such as opportunistic infections, damage of transplanted organs and secondary cancer [1, 2]. Therefore, new strategies to improve long-term graft survival while reducing/eliminating IS therapy and, ideally, inducing immunological tolerance are needed.

Cellular therapy can improve the outcome of solid organ transplantation through anti-inflammatory effects and the induction of immune tolerance. T cell-mediated immunomodulation is one of the main mechanisms to maintain operational tolerance (withdrawal of immunosuppressive drugs while maintaining normal graft function and histology) in vivo. It is now accepted that regulatory T cells (Tregs), a subpopulation of T helper lymphocytes, are responsible for this immunomodulatory activity, as a result of their suppressive effects directly on effector T cells and on antigen presenting cells [3, 4]. Circulating Tregs constitutively express CD25 and FoxP3 and represent 5–10% of all peripheral CD4+ T cells [5, 6]. Tregs, which are fundamental for the maintenance of immune homeostasis, demonstrated a key role for transplantation tolerance in animal models by impairing the function of CD8+ T cells [7–10]. In humans, cell therapy with human Tregs for the induction of transplantation tolerance represents a promising strategy [10, 11]. Indeed, clinical trials using this approach have already demonstrated that expanded polyclonal and antigen-specific Tregs are safe and effective in the treatment of GVHD and Type 1 diabetes [12–17]. Conversely, very few results have been published on clinical trials testing the efficacy of Tregs in solid organ transplantation [18, 19]. Moreover, isolated/ex vivo expanded Tregs have been only tested in animal models [20–22].

Here, we first established a preclinical protocol for expansion/isolation of Tregs from patients with end-stage liver or kidney disease being in the waiting list for liver/kidney transplantation (LT/KT). We then scaled up and optimized such protocol according to good manufacturing practice (GMP) to obtain high numbers of purified Tregs which were tested in vitro and in a xenogeneic acute graft-versus-host disease (GVHD) mouse model.

## Materials and methods

### Patients

Peripheral blood (20–60 mL) was obtained from 14 LT and 9 KT patients. Patients characteristics are outlined in Table 1. Patient selection for GMP Tregs isolation/expansion was based on the following inclusion criteria: (1) age ≥ 18 years; (2) diagnosis of end-stage kidney disease in waiting list for living donor kidney transplantation or diagnosis of end-stage liver disease in waiting list for liver transplantation. Exclusion criteria included: (1) HIV, HBV, HCV positivity; (2) syphilis antibody positivity; (3) combined transplant; (4) concurrent uncontrolled infection. Patient selection for in vitro experiments were as above described except virus positivity. Peripheral blood (20–60 mL) and buffy-coat (30 mL) were also obtained from age and sex matched healthy controls. Human studies were conducted in accordance with the Declaration of Helsinki and approved by the local ethical Committee (*232/2015/O/Tess by Investigation Drug Service*, *Azienda Ospedaliero*-*Universitaria di Bologna*). Informed consent was obtained from all the subject prior to enrollment into the study.

**Table 1:** Patients characteristics

	KT	LT	CTR
Total number	9	14	10
Sex (male/females)	3/6	8/6	5/5
Age (years; mean ± SD)	53 ± 13	61 ± 10	58 ± 12
Disease			
HCV/HBV liver cirrhosis	N = 0	N = 8	NA
Alcoholic liver cirrhosis	N = 0	N = 3	NA
Dysmetabolic liver cirrhosis	N = 0	N = 2	NA
Cryptogenetic liver cirrhosis	N = 0	N = 1	NA
Epatorenal polycystic disease	N = 4	N = 0	NA
End stage kidney disease	N = 4	N = 0	NA
Drug-induced tubulointerstitial nephritis	N = 1	N = 0	NA

*NA* not applicable, *LT* liver transplant, *KT* kidney transplant, *CTR* healthy control

### Circulating Treg enumeration

Enumeration by flow cytometry of circulating Treg (CD4+CD25+CD127−FoxP3+) was carried out in the peripheral blood (PB) of selected KT and LT patients (n = 7 and n = 10, respectively) and of healthy controls (n = 9). The conjugated monoclonal antibodies used are shown in Additional file 1: Table S1. Surface marker staining was performed for 15 min at room temperature. For intracellular staining, anti-human FoxP3 (PCH101) Staining Set PE Kit was used (eBiosciences), according to the manufacturer’s instructions. Isotype control rat IgG2 PE was used as a control. Briefly, cells were stained for surface markers CD4, CD25 and CD127, washed once in PBS and then fixed/permeabilized. After washing, cells were incubated with anti-human FoxP3 antibody for 30 min at 4 °C in the dark. A lysis buffer (Becton–Dickinson) was used in order to lysate red blood cells. The phenotype of Tregs was analyzed by flow cytometry FACSCantoII (Beckton Dickinson). Data were analyzed using the FACSDiva software (Becton–Dickinson). The percentage of positive cells was calculated by subtracting the value of the appropriate isotype controls. The absolute number of positive cells per µL was calculated as follows: percentage of positive cells × white blood cell count (WBC)/100.

### Tregs isolation and expansion

EDTA-anticoagulated peripheral blood (60 mL) was collected from 4 LT patients, 2 KT patients and buffy-coat (30 mL) from 5 controls. Peripheral blood mononuclear cells (PBMC) were then isolated by Ficoll-Hystopaque density gradient centrifugation.

Isolation: freshly isolated CD8−CD25+ T cells were purified from PBMC by negative selection of CD8+ T cells followed by positive selection of CD25+ T cells using specific Miltenyi-Biotec Beads (CD8 microbeads human and CD25 microbeads II human) with MidiMACS separator and a purity (CD4+CD25+) of > 90%.

Expansion: freshly isolated cells were plated at 1 × 10^6^/mL cells and activated with anti-CD3/CD28 coated beads (Invitrogen, Paisley, UK; Miltenyi Biotech) at a 4:1 bead:cell ratio at day 0 and then 1:1 bead:cell ratio weekly. Cells were expanded in culture media (TECSMacs GMP medium, Miltenyi Biotech) 5% human AB plasma containing rapamycin (100 nM) (Rapamune^®^, Wyeth, USA) for 21 days at 37 °C and 5% CO_2_. IL-2 (1000 IU/mL, Proleukin^®^, Novartis, UK) was added at day 4 post-activation and replenished every 2 days. Cells were restimulated with beads every 7 days. After 21 days of culture, beads were magnetically removed and the cells washed in TECSMacs GMP medium. After washings, fresh beads, rapamycin and IL-2 were added. Expanded cells were used for further analysis at each time of re-stimulation up until day 21 of expansion.

### Phenotypic characterization

Phenotypic characterization were performed on day 0, 7, 14, 21 of cultures for ex vivo expansion and after cryopreservation/thawing by flow cytometry as above described. Surface marker staining was performed in order to asses the content of Tregs and contaminant cells [monocytes (CD14+), B (CD19+) and T cells (CD8+), NK cells (CD56+), Th17 cells (CD196+CD161+)] present at each time point of culture (Additional file 1: Table S1).

### FOXP3 promoter demethylation analysis

The highly conserved Treg-specific demethylation region (TSDR) within the human *FOXP3* gene is demethylated exclusively on Tregs and not in any other blood-cell types. Specific DNA methylation of the Treg *FOXP3* is referred to intron 1, as previously identified [23]. DNA methylation of *FOXP3* gene (CNS2 region) of expanded cells (day + 21), either freshly isolated or after cryopreservation/thawing, was evaluated for a total of 6 subjects (3 patients: 1 KT and 2 LT patients; and 3 controls). Genomic DNA was extracted from purified Tregs using the *AllPrep* DNA/RNA Mini Kit (QIAGEN, Hilden, Germany). 500 ng of DNA was bisulphite-converted using the EZ DNA Methylation Kit (Zymo Research Corporation, Orange, CA) according to manufacturer’s instructions, except for the thermal conditions of the conversion (21 cycles of 55 °C for 15 min and 95 °C for 30 s). Bisulphite-treated DNA was eluted in 100 μL of water. DNA methylation analysis of the genomic region chrX:49,117,049–49,117,467 within *FOXP3* gene body was performed by EpiTYPER assay (Sequenom, San Diego, CA), a quantitative method based on mass spectrometry that allows to evaluate methylation level at single CpG sites/groups of adjacent CpG sites (CpG units). Ten ng of bisulphite-treated DNA were PCR-amplified and processed following manufacture’s instructions. The bisulphite specific primers were: *FOXP3* forward, AGGAAGAGAGATTTGTTTGGGGGTAGAGGATTTA; *FOXP3* reverse, CAGTAATACGACTCACTATAGGGAGAAGGCTCAAAAAAAACCAAATCTTCAAAACT.

For each gene, CpG sites with missing values in more than the 20% of the samples were removed, as well as samples with missing values in more than the 20% of CpG sites. The R package *massArray* was used to test if bisulphite conversion reaction run to completion [24]. For all samples analysed bisulphite conversion was from 98.9 to 100%.

### Mixed leukocyte reaction assay

The T cells were used as autologous responder cells for in vitro suppression assays. The suppressive activity of freshly isolated and expanded cells were tested by co-culturing CD8−CD25− T cells, labeled with 5 µM carboxyfluorescein succinimidyl ester (CFSE, Invitrogen), with serial dilutions of freshly isolated (CD8−CD25+ T cells) and ex vivo expanded autologous Tregs in the presence of CD3/CD28 GMP beads at ratio previously shown by us (in house experiments) to be effective in dose–response experiments (1:10 CD8−CD25− T cells to bead ratio) in RPMI-1640 medium containing 10% FBS, 1% penicilline/streptomycine and l-glutamine (1%) for 5 days at 37 °C and 5% CO_2_. Proliferation was analyzed by flow cytometry. The percentage of proliferative CD8−CD25− T cells in the absence of Tregs was taken as 100% proliferation.

### GMP Treg isolation and expansion for in vivo experiments

The GMP manufacturing was performed in the Cell Factory of Fondazione IRCCS Ca’ Granda Ospedale Maggiore Policlinico, certified in compliance with European GMP regulations by the Italian Drug Agency (authorization number aM-51/2018).

Steady state leukapheresis from 2 patients (1 KT and 1 LT patient) were processed using GMP-compliant devices and reagents (Additional file 1: Figure S1A). The subjects were evaluated for venous accesses suitability. Leukapheresis was carried out by the COM.TEC^®^ (Fresenius Kabi AG, Bad Homburg, Germany) cell separator. The treatment of two blood volumes was set up as the procedure end-point. ACD-A was used for anticoagulation at a ratio of 1:14–1:13. For prophylaxis of citrate-related hypocalcemia, calcium gluconate was administered intravenously during the leukapheresis.

CD8−CD25+ cells were purified using the CliniMACS System (Miltenyi Biotec, Bergisch Gladbach, Germany) according to GMP procedures and following manufacturer’s instructions. Briefly, we performed a sequential two-step process based on the magnetic depletion of CD8+ T cells, followed by a positive selection of CD25+ cells after an over night storage, using monoclonal antibodies anti-CD8 (Miltenyi Biotec) and anti-CD25 (Miltenyi Biotec), as described in the CliniMACS user manual for cell preparation, magnetic labelling, and selection.

Forty millions of CD8−CD25+ cells were expanded in vitro in gas-permeable culture bags (MACS GMP Cell Expansion Bags and MACS GMP Cell Differentiation Bags, Miltenyi Biotec) for 3 weeks in complete medium, consisting of TexMACS Medium (Miltenyi Biotec) supplemented with 100 nM rapamycin (Miltenyi Biotec), 5% allogeneic human heat-inactivated AB plasma, 500–1000 UI/mL IL-2 (Proleukin, Novartis Pharmaceuticals Canada Inc, Dorval, Quebec, Canada). On day 0, 7 and 14, anti-CD3/CD28 beads (MACS GMP ExpAct Treg Beads, Miltenyi Biotec) were added at different beads:cells ratio 4:1, 1:1, 1:1 respectively.

At day 21 all the cultured cells were collected and the beads were removed using the CliniMACS device, according to manufacturer’s instructions. Cells were counted by an automated and validated method (Nucleocounter System, Chemometec, Allerød, Denmark). The Treg markers were evaluated by flow cytometry (BD FACSCanto II, BD Bioscience, San Jose, CA, USA) using the following antibodies: CD45 APC-H7 (BD Bioscience), CD4 FITC (BD Bioscience), CD25 APC (BD Bioscience), CD127 PE-Cy7 (BD Bioscience) and FoxP3 PE (eBioscience, San Diego, CA, USA).

Negative fraction (CD8−CD25− T cells) after GMP selection at day 0 and the final product after GMP expansion at day 21, were cryopreserved and thawed as above described.

### Cryopreservation and thawing of freshly isolated and expanded cells

Freshly isolated fractions (CD8−CD25+ and CD8−CD25− T cells) and expanded cells (after 7, 14 and 21 days of expansion) were washed and cryopreserved with 10% DMSO (CRYOSERV, Mylan Institutionals, Canonsburg, PA, USA), 10% HSA (HAS; Kedrion, Lucca, Italy) in a saline solution (B. Braun, Melsungen, AG, Germany) using a controlled-rate freezer. The frozen units were transferred and stored immediately to vapor-phase liquid nitrogen into dedicated tanks. All the cellular fractions were thawed at 37 °C and the viability, phenotype and suppressive function were assessed as above described.

### Xenogeneic GVHD mouse model

NOD scid gamma (NSG) female mice (6–7 weeks of age) were purchased from Charles River (Calco, Italy). All animals were housed under specific pathogen-free conditions in compliance with guidelines of the San Raffaele Institutional Animal Care and Use Committee (IACUC number: 632). Mice were maintained for at least 5 days in the animal facility for acclimatization before transplantation. To promote the engraftment of transplanted cells injected intraperitoneally, all mice were conditioned with total body irradiation (1.75 cGy) on day 1. Of note, day 1 of the study is considered the day of transplant. All groups of mice, transplanted as well as control mice, were monitored for 7 weeks. Clinical signs of GVHD (e.g., hunched back, fur loss, skin inflammation) were monitored daily. Body weight was monitored throughout the duration of the study. Animals showing marked clinical signs as of distress, loss of weight equal to or greater than 20% of their starting weight were immediately sacrificed.

To establish whether the expanded Tregs could counteract acute GVHD, a xeno-GvHD model was induced by intraperitoneally transfer of human autologous CD8−CD25− T cells from 1 LT and 1 KT patient into mice. Both CD8−CD25− T cells and expanded Tregs were injected within 30 min from thawing (Additional file 1: Figure S1B). Specifically, human CD8−CD25− T cells (6 × 10^6^) from LT or KT patients were injected with (simultaneous injection) or without autologous Treg (6 × 10^6^) at a Treg: CD8−CD25− T cells ratio of 1:1. Timing of Treg infusion is crucial. In our model Tregs were infused at the time of T effector transplantation.

### In vivo engraftment monitoring

To assess the in vivo persistence of human cells, transplanted mice were bled 4 weeks after transplantation and sacrificed 7 weeks after transplantation. Phenotype analysis of injected cells and of peripheral blood at 4 weeks (± 3 days) after transplantation and of PB and spleen at 7 weeks (± 3 days) after transplantation was performed (Additional file 1: Figure S1C). Following a Fc blocking step, cell surface staining was performed with anti-mouse CD45 and anti-human CD4, CD3, CD25, FoxP3 mAbs at 1:100 dilution in staining buffer (PBS, 2% FCS, 0.1% NaN3) (list of mAbs is in Additional file 1: Table S1). To detect FoxP3, cells were treated and stained with the FoxP3 fixation/permeabilization kit according to the manufacturer’s instructions (eBioscience). Samples were acquired on a BD FACSCanto II flow cytometer. Manual analysis of flow cytometry data was performed with FCS Express V4 (DeNovo Software, Glendale, CA).

### Statistical analysis

Statistical analyses were performed using GraphPad Prism (GraphPad Prism 5.0 soft-ware, SanDiego CA, USA), and data are presented as mean ± standard error of the mean (SEM). For all experiments involving multiple comparisons, analysis of variance (ANOVA) followed by a Dunnett’s post hoc test was used. For those who involved comparisons between two groups, Student’s t-test was used. The level of significance was set at p ≤ 0.05.

## Results

### Enumeration of circulating Treg from LT and KT patients

We firstly enumerated circulating CD4+CD25+CD127−FoxP3+ Tregs in End-Stage Organ Disease patients and controls. The mean absolute numbers of circulating Tregs from LT (19.8 ± 10/mL) and KT (18.8 ± 7/mL) patients was not significantly different from that of healthy controls (17 ± 4/mL) (p = NS; Additional file 1: Figure S2).

### Tregs isolation, expansion, cryopreservation and thawing

**Fig. 1:**
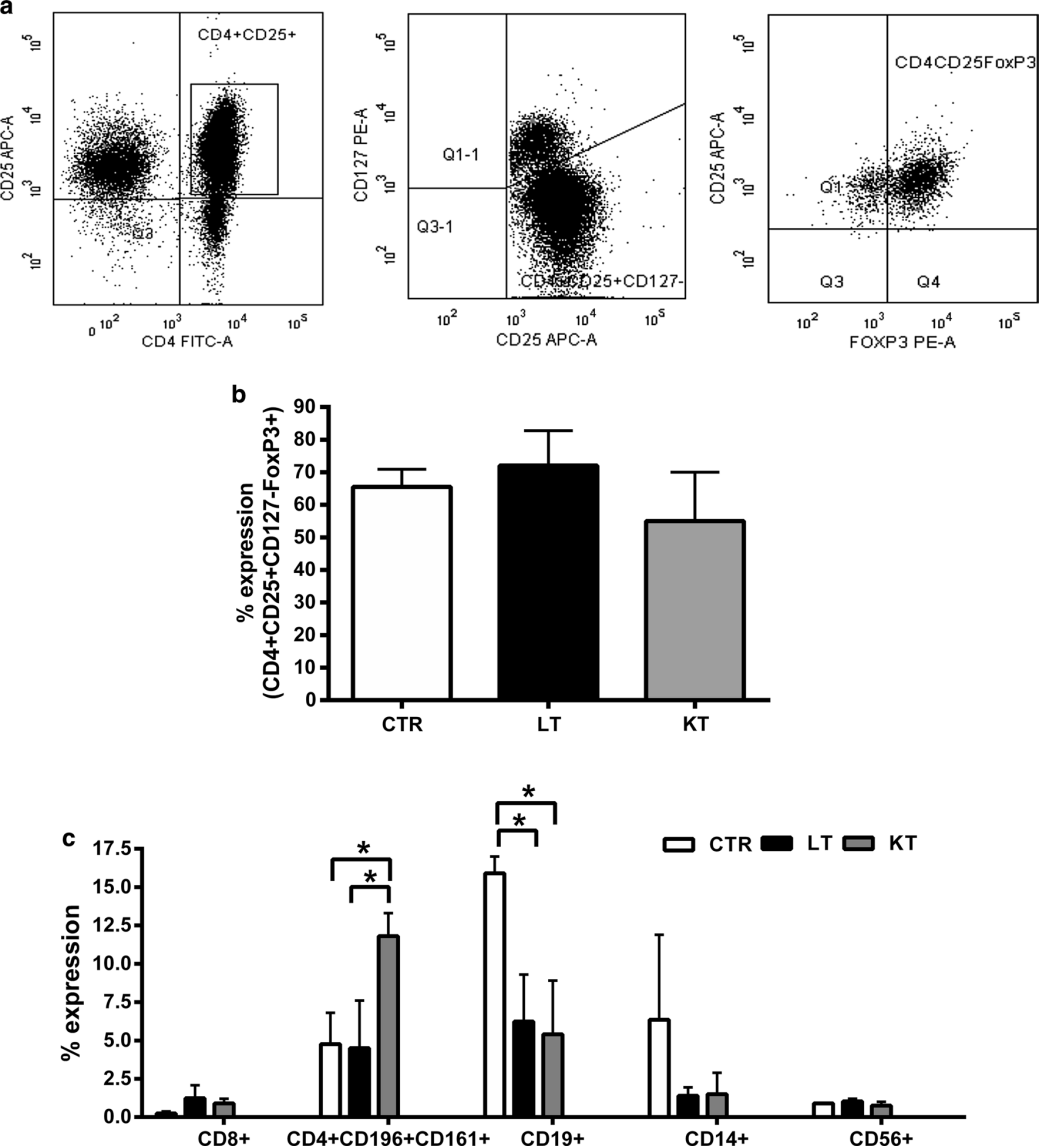
Phenotype of the freshly isolated Tregs. **a** The gating strategy of Tregs identification after CD8 depletion and CD25 enrichment is shown. Dot plots depict the expression of CD25, CD127 and FoxP3. Interestingly, the majority of the freshly isolated cells expressed CD25. Dot plots are representative of 11 independent experiments from patients/controls. **b** Graph shows Treg purity, as percentage of CD4+CD25+CD127−FoxP3+ cells after isolation from PB of CTR, LT and KT patients. No significant difference was observed between patients and controls (p = NS). **c** Graph displays the percentages of contaminant cells in the freshly isolated product from CTR, LT and KT patients. Data are represented as mean ± SEM (*p < 0.05)

Starting from 60 mL of PB (patients) and 30 mL of buffy-coat (healthy controls), CD8 depletion with subsequent CD25+ enrichment of PBMCs yielded a median of 3.7 × 10^6^ nucleated cells (range 1.28–8.8 × 10^6^) in healthy controls, 1.47 × 10^6^ nucleated cells (range 5.3 × 10^5^–2.56 × 10^6^) in LT patients and 1.77 × 10^6^ nucleated cells (range 8.5 × 10^5^–2.7 × 10^6^) in KT patients. The freshly isolated CD8−CD25+ T cells were 2.0 ± 1.2, 1.9 ± 1.3 and 2.6% ± 0.7 of the PBMCs, respectively, with no significant differences between patients/controls (p = NS) (data not shown). The Fig. 1a shows the gating strategy for identification of CD4+CD25+CD127−FoxP3+ Tregs which represented more than 80% of the cells (range 70–96%) within the CD8−CD25+ population. The median Treg purity (CD4+CD25+CD127−FoxP3+ cells) was 72% (range 50–84) and 55% (range 40–70) in LT and KT patients, respectively and 65% (range 54–80) in healthy controls (Fig. 1b). No significant difference was observed between patients and controls (p = NS). On day 0, contaminating cells in healthy controls were CD19+ B cells (16%, range 14.8–17), Th17 cells (4.7%, range 0.7–6.9) and CD14+ monocytes (6.3%, range 0.8–11.4). In LT and KT patients, contaminating cells were mainly CD19+ B cells (6.8%, range 1.1–8.4; 5.4%, range 1.9–8.9, respectively) and Th17 cells (7.3%, range 1.4–12.8; 11.8%, range 10.3–13.3, respectively). Natural Killer cells and monocytes content was always very low (below 1%) (Fig. 1c). By comparing patients and controls, Th17 cells were significantly increased in KT cells as compared with LT or healthy controls (p ≤ 0.05 vs. LT patients; p ≤ 0.05 vs. healthy controls). In addition, CD19+ cells were significantly reduced in both patient groups as compared with the normal counterparts (p ≤ 0.05 for LT patients; p ≤ 0.05 for KT patients).

**Fig. 2:**
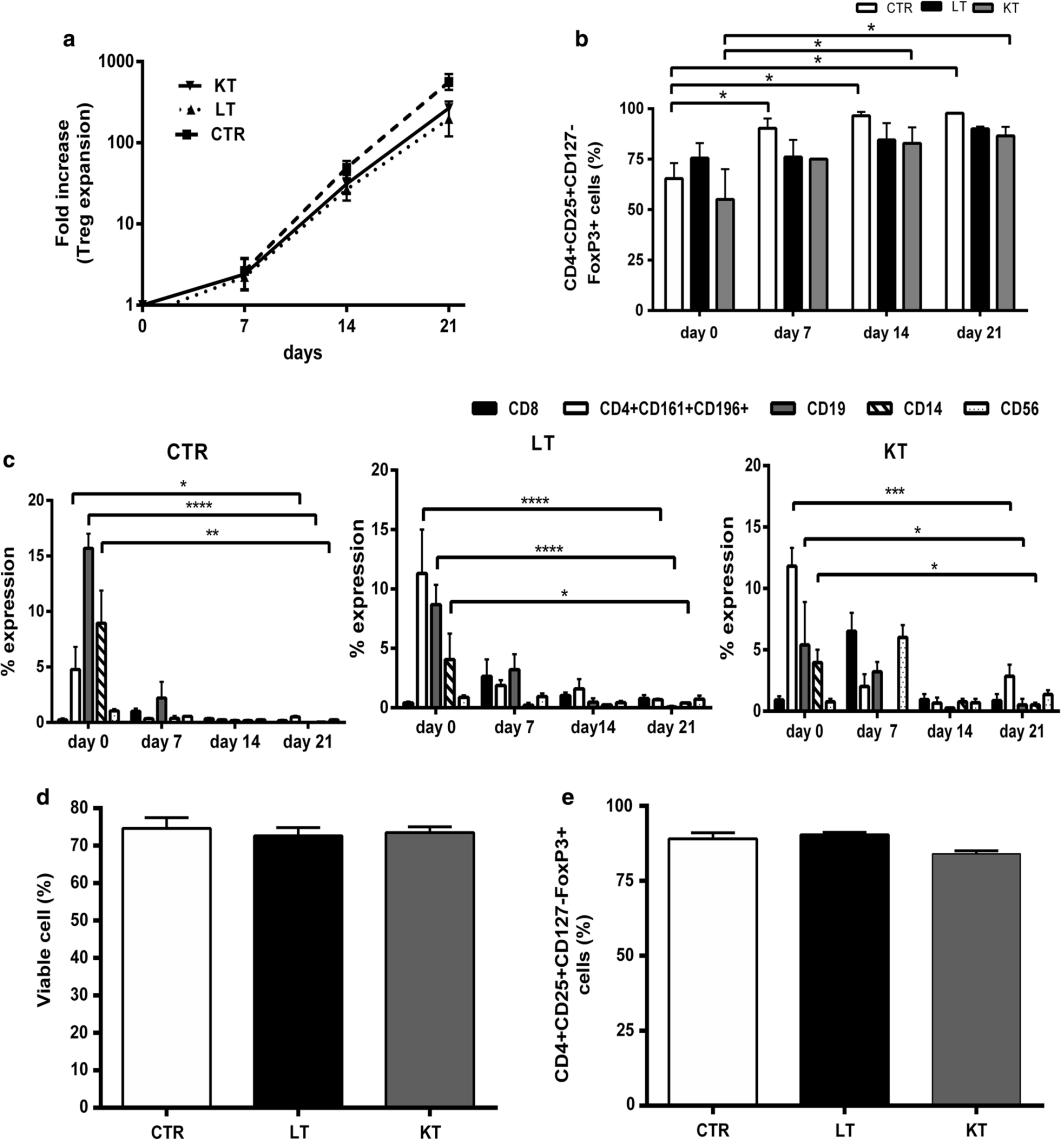
Phenotype of the in vitro expanded Tregs. **a** Tregs from CTR, LT and KT patients were expanded up to 21 days with IL-2 and rapamycin. Fold expansion of day 7, 14 and 21 was calculated as compared to day 0 seeded cells. **b** Graph shows the percentage of Treg (CD4+CD25+CD127−FoxP3+) at each time point stimulation. Treg purity increased significantly during expansion in CTR and KT patients. **c** The graph shows the percentages of contaminant cells during the expansion period. The contaminant cells decreased below 1% in CTR and in LT patients and below 3% in KT patients at the end of expansion. **d** Viability of cryopreserved/thawed day 21 Tregs is shown. No significant difference was observed between patients and CTR (p = NS). **e** Phenotype of the cryopreserved/thawed day 21 Tregs is shown as mean percentages of CD4+CD25+CD127−FoxP3+ cells. The thawed Tregs were almost all FoxP3 positive in patients/CTR. No significant difference was observed between patients and controls (p = NS). Data are represented as mean ± SEM (*p < 0.05)

After 21 days, Tregs expanded a median of 197-folds (range 26–567) in LT patients, 266-folds (range 233–300) in KT patients and 559-folds (range 342–826) in healthy controls (Fig. 2a). Differences between patients and controls were not statistically significant. Starting from freshly isolated 500 × 10^3^ cells, the final yield was a median of 255 × 10^6^ (range 171 × 10^6^–413 × 10^6^) nucleated cells in healthy controls, 48 × 10^6^ (range 30 × 10^6^–284 × 10^6^) nucleated cells in LT patients and 133 (range 116 × 10^6^–150 × 10^6^) nucleated cells in KT patients (data not shown). Treg purity (CD4+CD25+CD127−FoxP3+) increased significantly during expansion (Fig. 2b) in healthy donors and KT patients (p ≤ 0.05). Contaminating cells, namely B cells, Th17 cells and monocytes decreased below 1% in healthy controls and LT patients and below 3% in KT patients (Fig. 2c).

When cryopreserved/thawed 21 days expanded Tregs were analyzed, the mean percentage of viable cells was always more than 75% with no significant differences between patients and controls (Fig. 2d). The mean percentage of CD4+CD25+CD127−FoxP3+ cells was always > 80% (Fig. 2e).

**Fig. 3:**
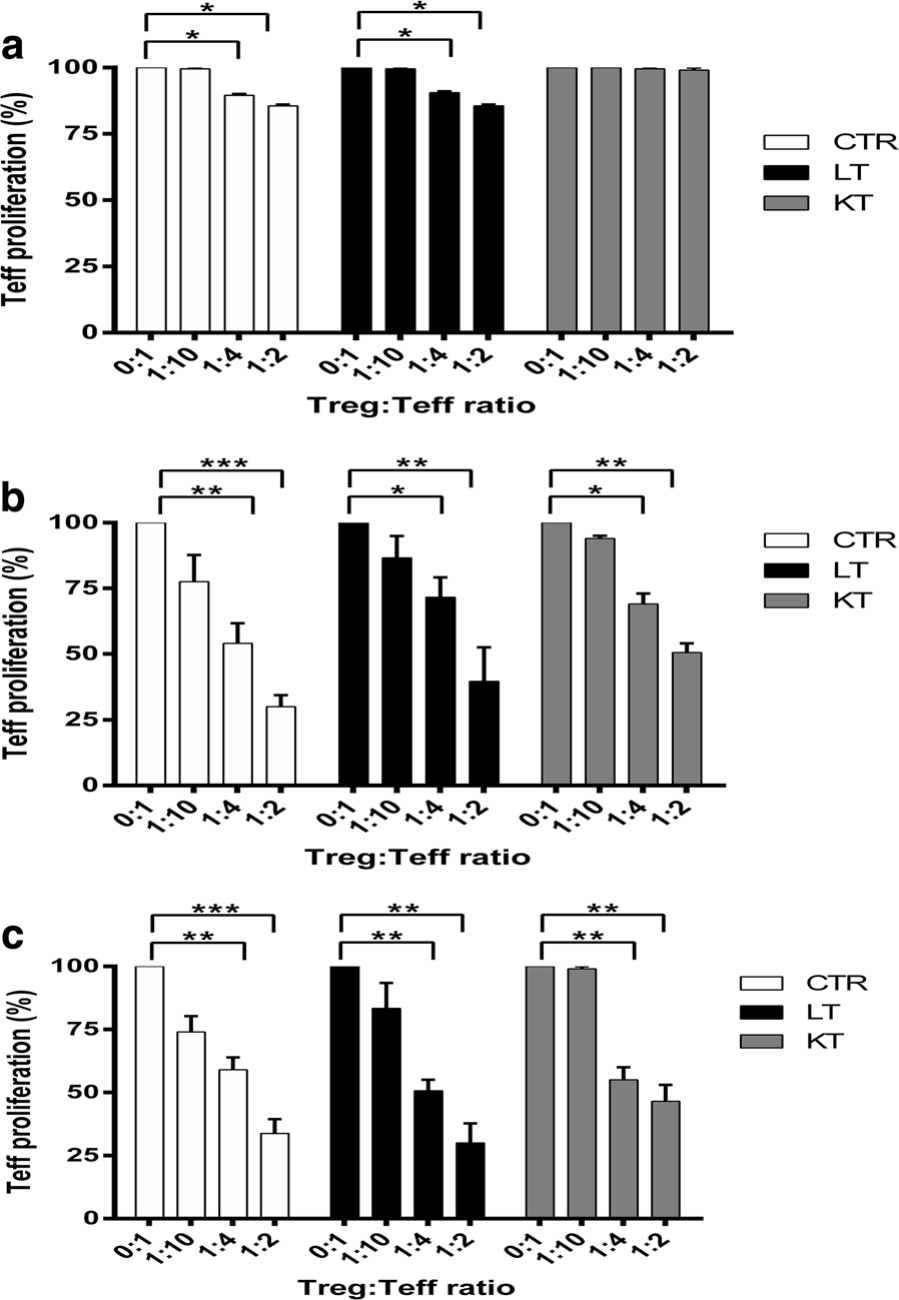
Suppressive function of the in vitro expanded Tregs. The graphs show the mean percentages of proliferation of the CFSE-labelled autologous CD8−CD25− T cells in the presence of CD3/CD28 GMP beads and Tregs at different ratios. **a** MLR with freshly isolated Tregs (day 0). Freshly isolated Tregs from the three groups were limited (CTR and LT patients) or very poorly (KT patients) suppressive. **b** MLR with freshly isolated expanded Tregs (day 21). Expanded Tregs from patients/CTR had significant suppressive activity at 1:2 and 1:4 (Treg:CD8−CD25− T cells) ratio. **c** MLR with expanded and cryopreserved/thawed day 21 Tregs. Cryopreservation/thawing did not significantly affects the suppressive activity of day 21 expanded Tregs of patients/CTR. Data are represented as mean ± SEM (*p < 0.05, **p < 0.01, ***p < 0.001)

When functional assays were performed, freshly isolated cells (CD8−CD25+ T cells) from the three groups exerted a limited (healthy controls and LT patients) or poor (KT patients) suppressive effect toward autologous CD8−CD25− T cells proliferation in vitro (Fig. 3a). After 21 days of in vitro expansion, flow cytometry analysis demonstrated that, according to the increased T regulatory phenotype, Tregs from patients/controls had significant suppressive activity (Fig. 3b), which was not modified by cryopreservation/thawing (Fig. 3c).

All together these data demonstrate that our small scale Treg isolation and expansion protocol from LT and KT patients lead to functional Tregs which are suitable for cell therapy. Moreover, cryopreservation/thawing does not impair phenotype/function of the expanded Tregs.

### FOXP3 methylation analysis

**Fig. 4:**
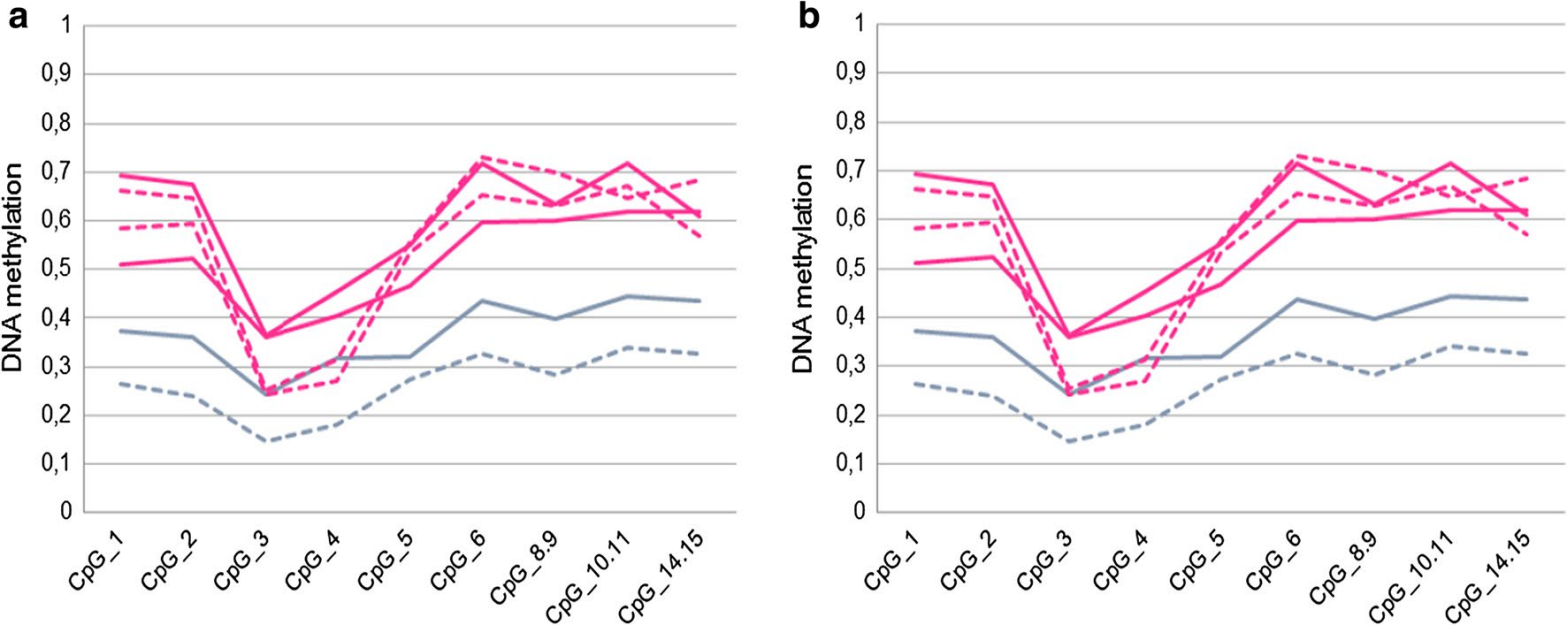
DNA methylation analysis of FOXP3 intron 1. **a** DNA methylation values of *FOXP3* intron 1 in day + 21 freshly isolated GMP expanded Tregs from patients/healthy controls. **b** Differences in DNA methylation values of *FOXP3* locus between day + 21 freshly isolated and day + 21 cryopreserved/thawed GMP expanded Tregs. In both the panels blue and pink lines correspond to male and female subjects respectively, while continuous and dashed lines correspond to healthy controls and patients respectively

Figure 4a shows that in males the locus, which is localized on X chromosome, displayed low DNA methylation values (CpG sites ranging from 0.08 to 0.44). As expected, in females CpG sites had higher methylation values (ranging from 0.24 to 0.73) due to X chromosome inactivation We evaluated DNA methylation differences at day + 21 in freshly isolated and cryopreserved/thawed expanded cells in patients and controls separately using paired t-test. The large majority of CpG sites was not significant, and intra-individual difference between the two groups were generally small, spanning between − 0.13 and 0.23. The only exception was the CpG_3 among controls (p-value: 0.002), but, also in this case, the difference between the freshly isolated and the cryopreserved/thawed cells was small, ranging from 0.06 to 0.07 (Fig. 4b).

Collectively, these data suggest that the DNA methylation pattern of *FOXP3* gene is substantially conserved after cryopreservation/thawing of ex vivo expanded Tregs from LT and KT patients.

### In vivo study

To investigate whether GMP expanded Tregs from 1 KT and 1 LT patient have inhibitory function in vivo after thawing, we used a xenograft model of lethal GVHD. Specifically, irradiated NSG mice were injected with cryopreserved/thawed CD8−CD25− T cells either alone or in combination with autologous expanded cryopreserved/thawed Tregs at 1:1 ratio to assess their ability to ameliorate GVHD (Additional file 1: Figure S1). The rationale for this design is that early infusion of Tregs allows the cells to prevent T effector activation. After the immunomagnetic selection and expansion of 40 millions of freshly isolated CD8−CD25+ cells under GMP condition, we obtained 4.3 × 10^9^ total cells (107.1-fold expansion) for the KT patient and 4.6 × 10^9^ total cells (115.2-fold expansion) for the LT patient. Before expansion the percentage of CD45+/CD4+/CD25+ cells was 84.9% and 53.9% in KT and LT patient whereas, at the end of expansion, the percentage of CD45+/CD4+/CD25+ cells increased up to 98.3% and 89.2% respectively. At day 21 CD45+/CD4+/CD25+/CD127−/FoxP3+ cells were 79.2% and 84.4% respectively (data not shown).

**Fig. 5:**
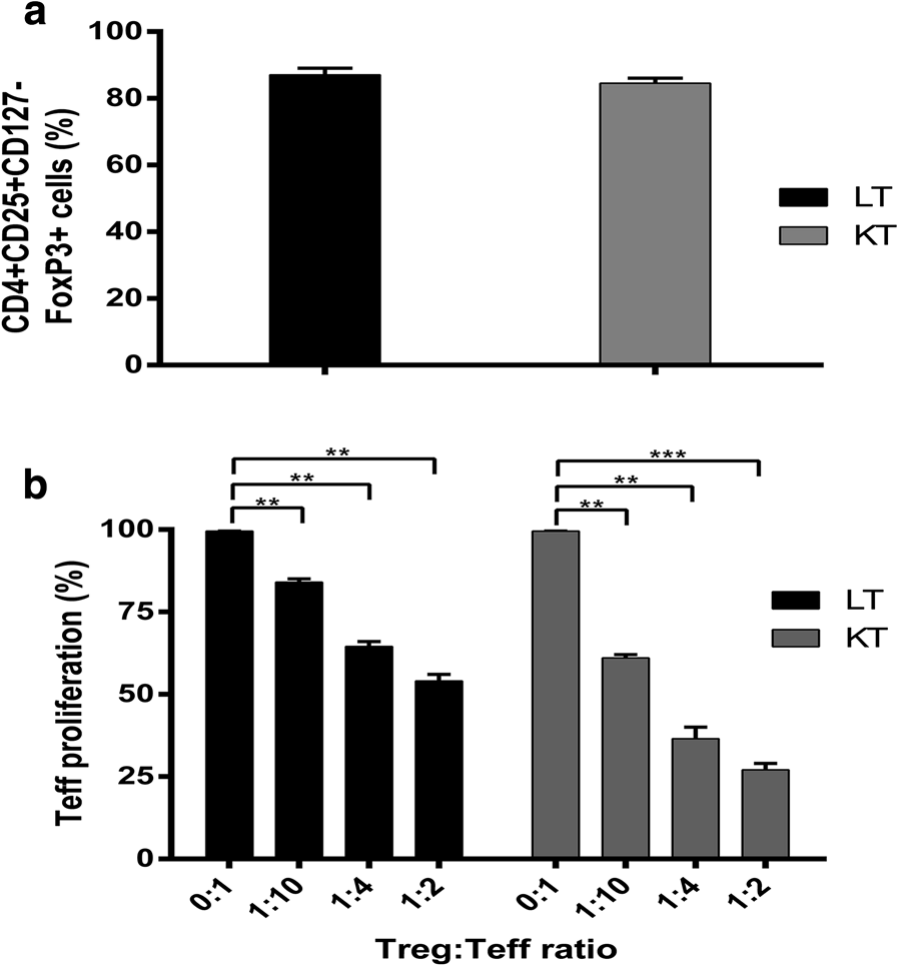
GMP Treg expansion for the in vivo study: phenotype and function of cryopreserved/thawed day 21 expanded Tregs. **a** Graph shows the percentages of CD4+CD25+CD127−FoxP3+ Tregs of the LT and KT patient after thawing. Almost all cells were FoxP3 positive. **b** MLR assay is shown. Expanded Tregs from the LT or KT patient had significant suppressive activity at 1:2 and 1:4 ratios. (**p < 0.01)

Tregs were then thawed and characterized for the phenotype and in vitro function. As shown in Fig. 5a, b, the thawed Tregs were more than 80% CD4+CD25+CD127−FoxP3+ and highly suppressive.

**Fig. 6:**
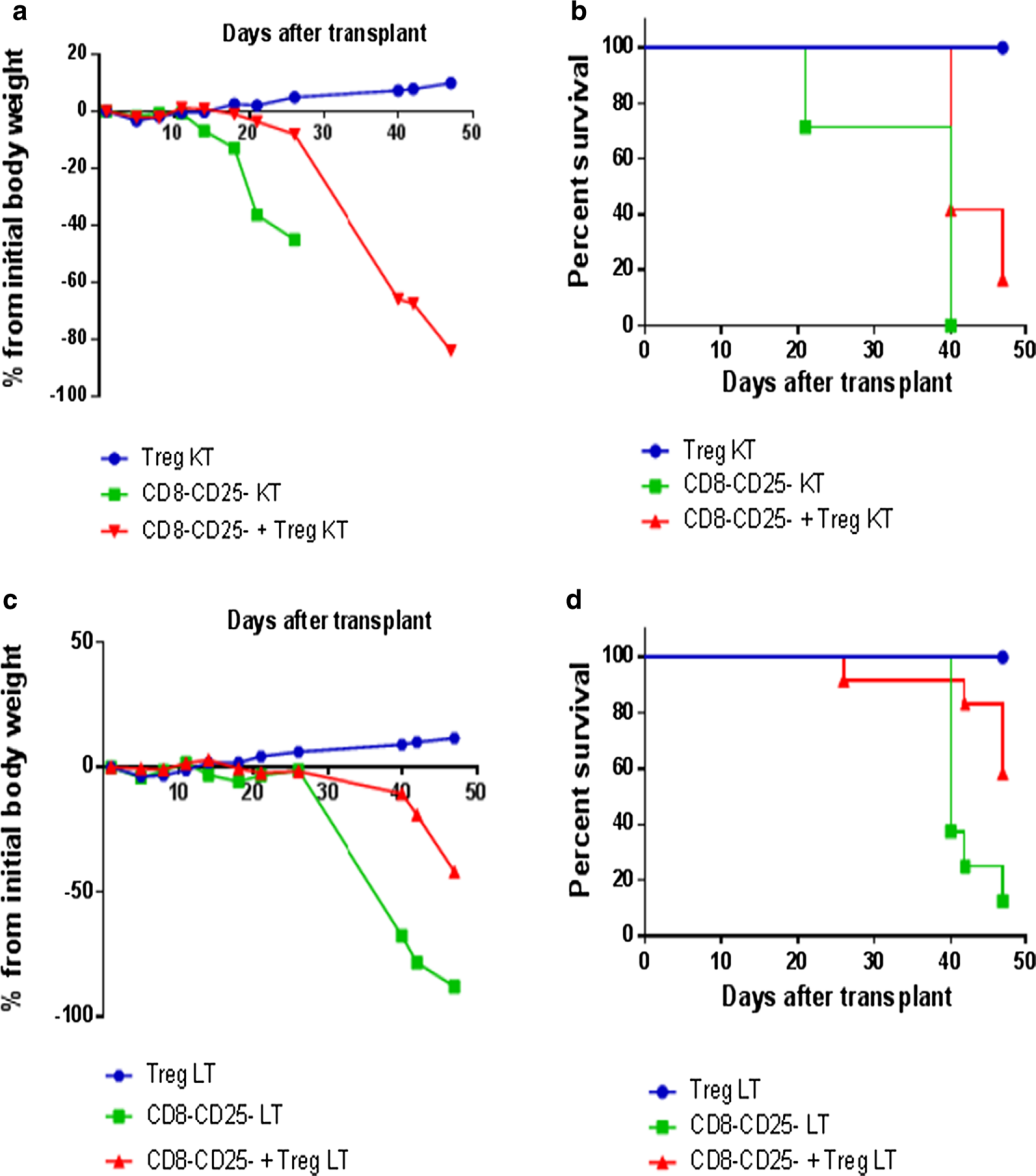
In vivo study: injection of cryopreserved/thawed GMP day 21 Tregs from 1 KT and 1 LT patient in a xenogeneic GVHD murine model. **a**, **c** Show the percentage of body weight variation of mice receiving CD8−CD25− T cells only, GMP Tregs only or GMP Tregs plus CD8−CD25− T cells from KT and LT patients, respectively. Data are presented as means (KT/LT: CD8−CD25− T cells n = 7/8 mice; GMP Tregs n = 6/6 mice; GMP Tregs plus KT CD8−CD25− T cells n = 12/12 mice). **b**, **d** Show the survival rate of mice receiving CD8−CD25− T cells only, GMP Tregs only or GMP Tregs plus KT CD8−CD25− T cells from KT and LT patients, respectively. [Survival of mice receiving KT cells: statistically significant differences were observed only for Tregs versus CD8−CD25+ cells (p < 0.001) and Tregs versus CD8−CD25+cells+ Tregs (p < 0.01); survival of mice receiving LT cells: statistically significant differences were observed only for Tregs versus CD8−CD25+ cells (p < 0.05)]. Overall, mice receiving GMP Tregs plus CD8−CD25− T cells showed a trend toward a time delay in body weight loss and survival as compared with mice injected with CD8−CD25− T cells only

Mice receiving KT CD8−CD25− T cells lost weight and all succumbed to GVHD by day 40. In contrast, mice receiving KT CD8−CD25− T cells and autologous Tregs showed delayed disease-associated weight loss and slightly increased survival rate (by day 48 17% of mice receiving CD8−CD25− T cells with Tregs from the KT patient survived versus 0% for mice receiving CD8−CD25− T cells only; p = NS) (Fig. 6a, b). Similar results were obtained with cells from the LT patient: by day 48 58% of mice receiving CD8−CD25− T cells with Tregs from the LT patient survived versus 12.5% for mice receiving CD8−CD25− T cells only (p = NS; Fig. 6c, d).

**Fig. 7:**
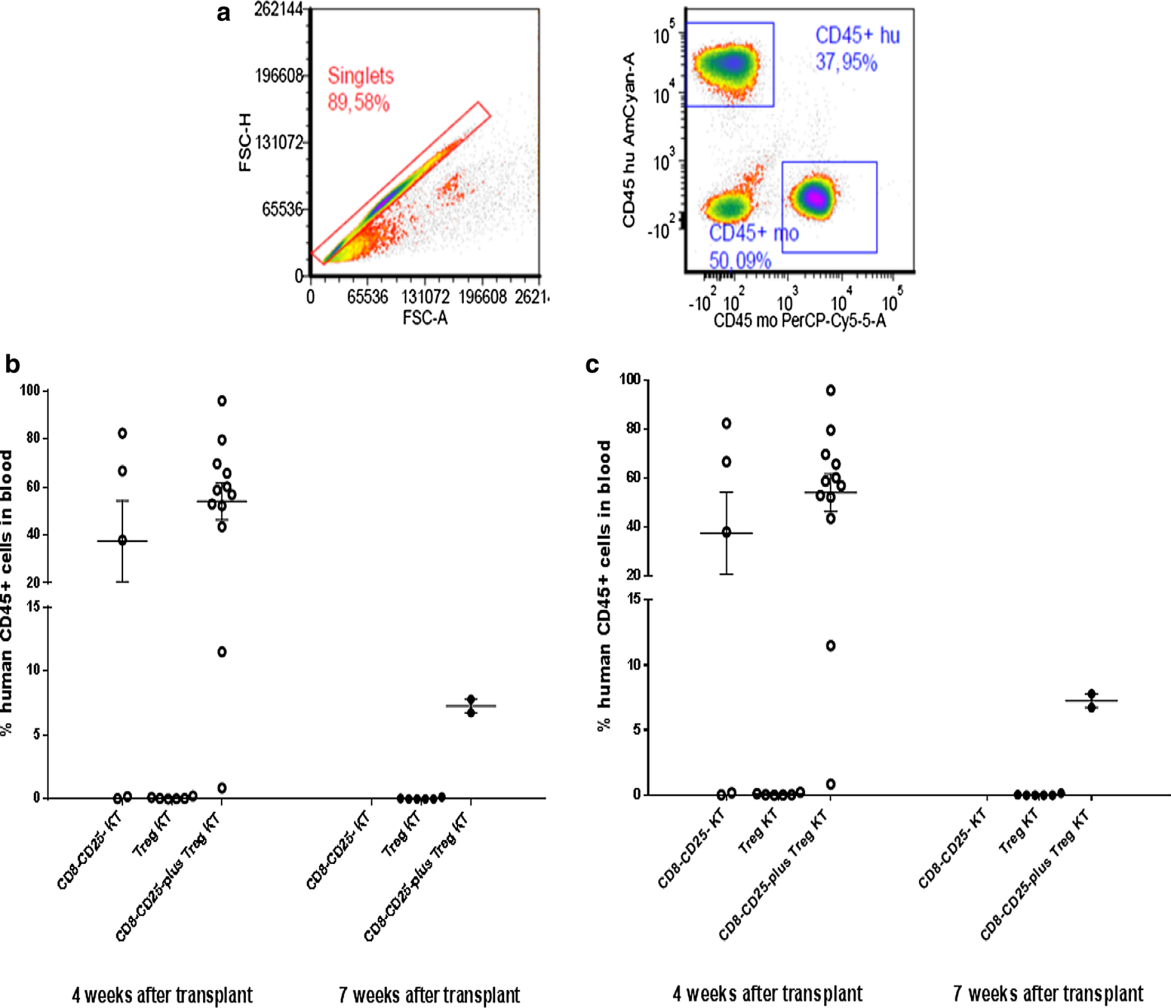
In vivo study: human CD45+ cells engraftment in the xenogeneic GVHD murine model. Animals were bled after 4/7 weeks and the percentages of human CD45+ cells in blood was determined by flow cytometry. **a** Representative dot plots of human CD45+ cell engraftment at 4 weeks after transplant are shown. **b**, **c** No human CD45+ cells were identified in the PB of mice after infusion of Tregs alone from the KT or LT patient. Conversely, a wide distribution of human CD45+ cells were observed in the PB of mice after transplant of KT/LT CD8−CD25− T cells alone or CD8−CD25− T cells plus Treg infusion. In addition to individual data, mean values and SEM are shown

To assess human cells engraftment in PB, animals were bled after 4/7 weeks and the percentages of human CD45+ cells in blood was determined (Fig. 7). The gating strategy is shown in Fig. 7a. No human CD45+ cells were identified in the PB of mice after infusion of Tregs alone from KT or LT patients. Conversely, a wide distribution of human CD45+ cells was observed in the PB of mice after transplant of CD8−CD25− T cells alone or CD8−CD25− T cells plus Treg infusion (Fig. 7b, c).

**Fig. 8:**
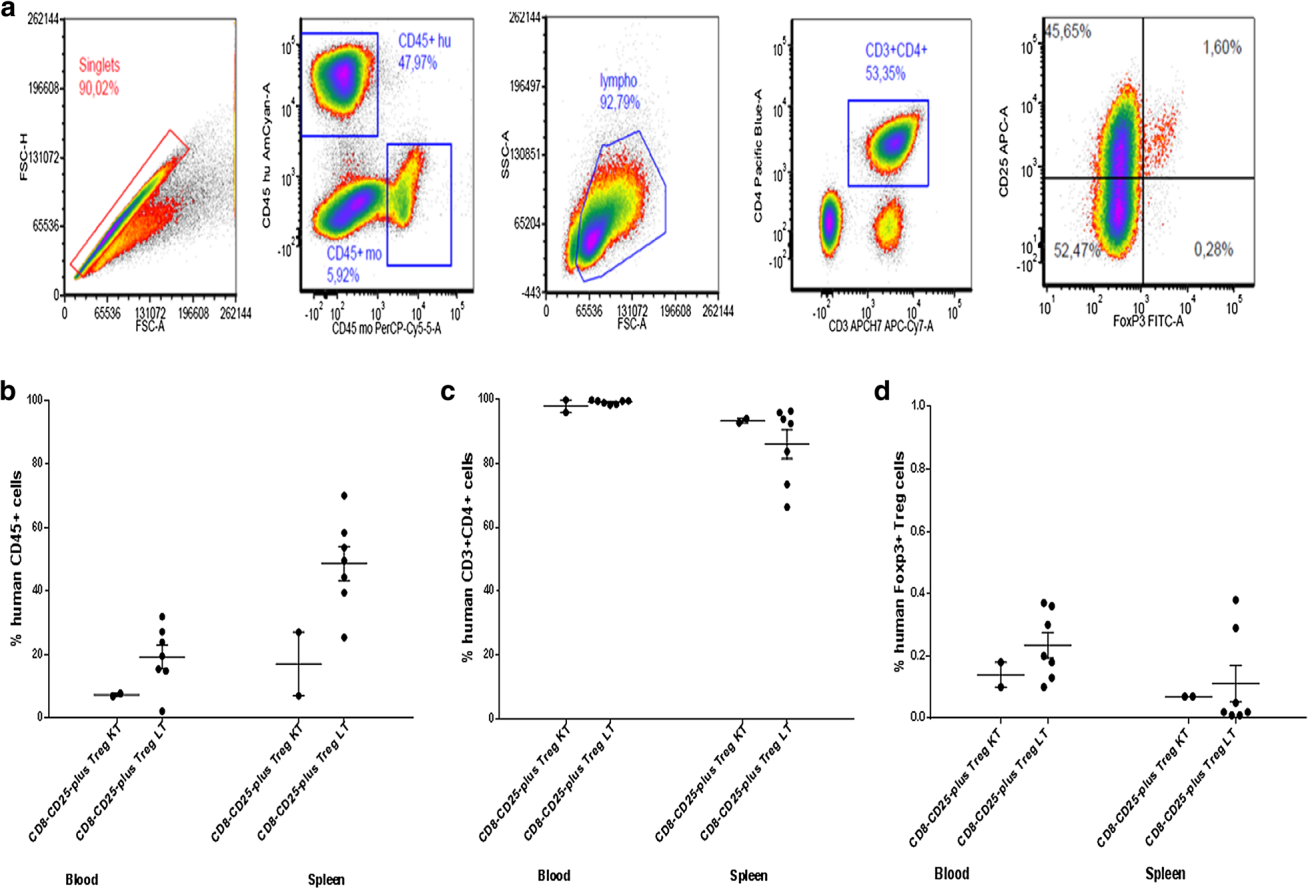
In vivo study: biodistribution of the infused GMP Tregs. Suspensions of spleen and PB after 7 weeks from transplant were stained with anti-human CD45, CD3, CD4, FoxP3 monoclonal antibodies and flow cytometry analysis was performed. **a** Depicts the gating strategy to identify the selected subpopulations. **b**–**d** Show the percentages of human CD45+, CD3+CD4+ and FoxP3+ Treg cells in the blood and spleen of mice after transplant of KT/LT CD8−CD25− T cells plus Treg. In addition to individual data, mean values and SEM are shown

To assess the biodistribution of infused human cells, spleen and PB cells of mice were stained with anti-human CD45, CD3, CD4, FoxP3 monoclonal antibodies after 7 weeks from transplant. The gating strategy is shown in Fig. 8a. By comparing PB and spleen, a higher mean percentage of human CD45+ cells was observed in the spleen versus PB of the mice after receiving CD8−CD25− T cells plus Treg of LT patients (p < 0.003; Fig. 8b). Of note, the majority of the human CD45+ cells were CD3+CD4+ in the blood/spleen of mice after receiving KT or LT cells (Fig. 8c). Human FoxP3+ Tregs were identified in the CD3+CD4+ fraction of PB and spleen of the two groups of mice without differences between KT and LT cells (Fig. 8d).

## Discussion

The use of Tregs for the treatment of GVHD and for inducing operational tolerance after solid organ transplantation is a promising approach.

Here, we isolated, expanded and functionally characterized Tregs from patients with end stage kidney and liver disease, with the aim to establish a suitable method to obtain immunomodulatory cells for adoptive immunotherapy post-transplantation.

We demonstrated that fully functional Tregs from patients in waiting list for solid organ transplantation can be isolated/expanded for clinical studies. These cellular products show homogenous regulatory T cells characteristics with very low percentage of contaminating cells. Therefore, unwanted immune side effects are minimized. Of note, based on phenotype/function, the isolated/expanded Tregs from KT and LT patients are similar. Interestingly, our cryopreservation/thawing procedure allowed cell suspensions with phenotype/function and hypomethylation of the *FOXP3* gene superimposable to that of the freshly isolated counterparts. This finding challenges the notion that cryopreservation of Tregs products is detrimental for their function [25, 26].

Our freshly isolated cells from healthy donors and LT/KT patients showed limited suppressive activity. This is in contrast with previous findings demonstrating 60–80% of suppressive activity by freshly isolated healthy donors cells, which were CD8−CD19−CD25+ cells [22] or CD8−CD25+ cells [27]. However, our results may be due (1) to the low purity of our freshly isolated cell suspension (CD8−CD25+ cells) and (2) to the fact that while our suppressive activity results were based on a 1:2 Treg/CD8−CD25− T cells working ratio, their ratio was 1:1 Treg/Teff. Whether this results is also due to the fact that our freshly isolated healthy donors cells derive from 24 h aged buffy-coats remains a matter of speculation.

Safinia et al. [10] firstly proposed a GMP production protocol to expand CD25+-enriched cells from PB in the presence of IL-2 and rapamycin to induce tolerance after liver transplantation. In their 36 days expansion protocol, multiple round of in vitro Treg stimulation were necessary to achieve clinically relevant Tregs number. In our protocol indeed, we obtained fully functional Tregs after 21 days of expansion containing less than 1% of contaminating CD8+ effector cells. Expanded, cryopreserved and thawed Tregs remain hypomethylated at intron 1 of the *FOXP3* locus, confirming their epigenetic stability. Functionally, Tregs showed suppressive function against autologous CD8−CD25− T cells.

Several human Treg products have been tested in animal models. However, previous data of Treg cell infusion in non human primates report conflicts regarding the ability of the infused cells to induce transplant tolerance [28–30]. Thus, we decided to use a mouse model of GVHD in immunodepressed mice [i.e. NOD-SCID-gamma KO (NSG) mice] receiving human effector T cells with or without GMP Tregs to prevent the onset of xenogeneic GVHD. In our mouse model, GMP Tregs from LT or KT patients proved to be safe and showed a trend toward reduced lethality of acute GVHD in vivo. Interestingly, GMP Tregs from LT and KT patients did not induce xenogenic GVHD and did not expand as documented by the absence of Tregs in the PB and spleen of mice after 4 and 7 weeks from transplantation. This is probably due to the lack of human IL-2.

Nevertheless, our data are consistent with previous models of xenogeneic GVHD and Treg infusion. The addition of CD25 expressing cells to human PBMC showed the amelioration of xenogeneic GVHD, whereas the depletion of all CD25+ cells led to the development of lethal xenogeneic GVHD [31]. Also, in vitro expanded Tregs from human peripheral blood or cord blood were able to ameliorate or suppress xenogeneic GVHD [32, 33]. More recently, it has been reported that the infusion of polyclonal human Tregs improved murine xenogeneic chronic GVHD [34]. In addition, Del Papa et al. [22] used a similar methodology (immune-magnetic Treg isolation and polyclonal expansion in the presence of CD3/CD28 beads, IL-2, and rapamycin for 19 days) achieving a median of 8.5-fold expansion and maintaining FoxP3 expression over the culture period. NSG mice that received human leukemic cells and expanded Tregs with conventional T cells were rescued from leukemia and survived without GVHD. Mice that received leukemic cells plus conventional T cells died of severe GVHD within 70 days.

## Conclusion

In summary, we demonstrated that expanded/thawed GMP Tregs from two different groups of patients with end-stage organ failure were fully functional in vitro and their infusion was safe and resulted in a trend toward reduced lethality of acute GVHD in vivo. These data further support the expanded/thawed Tregs-based adoptive immunotherapy in solid organ transplantation.

## Additional file

**Additional file 1: Table S1.** Antibodies for flow cytometry. **Figure S1.** Design of the in vivo study. **A.** Steady state leukapheresis from 2 patients (1 KT and 1 LT patient) were processed using GMP-compliant devices and reagents. Tregs positive fraction (CD8−CD25+ cells) was purified using the CliniMACS System. Forty millions of CD8−CD25 + cells were expanded in vitro for 3 weeks. At day 21 all the cultured cells were collected and the beads were removed using the CliniMACS device, according to manufacturer’s instructions. Negative fraction (CD8−CD25− T cells) after GMP selection at day 0 and the final product after GMP expansion at day 21 were cryopreserved and thawed as described in “Materials and methods” section. **B.** Irradiated NSG mice were infused with the KT or the LT CD8−CD25− T cells, either alone or in combination with autologous expanded Tregs at 1:1 ratio, to assess their ability to ameliorate GVHD. **C.** Mice were bled 4/7 weeks after transplantation and sacrificed 7 weeks after transplantation. FACS analysis of the injected cells (day 1), of PB (4 weeks ± 3 days after transplantation) and of PB and spleen (7 weeks ± 3 days after transplantation) was performed. **Figure S2.** Circulating Tregs in KT and LT patients. Mean absolute number of circulating CD4+CD25+CD127−FoxP3+ Tregs from healthy controls and selected LT and KT patients (p = NS).

## Data Availability

All data generated or analysed during this study are included in this article and its Additional file

## Abbreviations

Tregs: regulatory T cells
KT: kidney transplantation
LT: liver transplantation
GMP: good manufacturing practice
aGVHD: acute graft-versus-host disease
GVHD: graft-versus-host disease
IS: immunosuppressive
PB: peripheral blood
WBC: white blood cell
EDTA: ethylenediaminetetraacetic acid
PBMC: peripheral blood mononuclear cells
TSDR: Treg-specific demethylation region
CFSE: carboxyfluorescein succinimidyl ester
RPMI: Roswell Park Memorial Institute
FBS: fetal bovine serum
ACD: acid citrate dextrose
DMSO: dimethyl sulfoxide
HSA: human serum albumin
NSG: NOD scid gamma
