# Supplementary material for: Regulatory T cells from patients with end-stage organ disease can be isolated, expanded and cryopreserved according good manufacturing practice improving their function
Source: J Transl Med. 2019 Aug 5;17:250. doi: 10.1186/s12967-019-2004-2 (PMC6683529; doi:10.1186/s12967-019-2004-2)
Supplement: Supplementary file 1 — Additional file 1: Table S1. Antibodies for flow cytometry. Figure S1. Design of the in vivo study. A. Steady state leukapheresis from 2 patients (1 KT and 1 LT patient) were processed using GMP-compliant devices and reagents. Tregs positive fraction (CD8−CD25+ cells) was purified using the CliniMACS System. Forty millions of CD8−CD25 + cells were expanded in vitro for 3 weeks. At day 21 all the cultured cells were collected and the beads were removed using the CliniMACS device, according to manufacturer’s instructions. Negative fraction (CD8−CD25− T cells) after GMP selection at day 0 and the final product after GMP expansion at day 21 were cryopreserved and thawed as described in “Materials and methods” section. B. Irradiated NSG mice were infused with the KT or the LT CD8−CD25− T cells, either alone or in combination with autologous expanded Tregs at 1:1 ratio, to assess their ability to ameliorate GVHD. C. Mice were bled 4/7 weeks after transplantation and sacrificed 7 weeks after transplantation. FACS analysis of the injected cells (day 1), of PB (4 weeks ± 3 days after transplantation) and of PB and spleen (7 weeks ± 3 days after transplantation) was performed. Figure S2. Circulating Tregs in KT and LT patients. Mean absolute number of circulating CD4+CD25+CD127−FoxP3+ Tregs from healthy controls and selected LT and KT patients (p = NS). [file 12967_2019_2004_MOESM1_ESM.doc]

**Table S1.** Antibodies for flow cytometry

| Treg (CD4+CD25+CD127-CD45+Foxp3+) | CD4-FITC (SK3), CD25-PeCy7 (2A3), CD127-PE/APC (hIL-/R-M21), CD45-PerCpCy5.5 (2D1), from BD-Biosciences; CD25-Biotin (4E325) and Biotin-APC from Miltenyi; FoxP3-PE (PCH101) from eBioscience. |
| --- | --- |
| Contaminant cells (T cells, B cells, NK cells, Th17 cells, monocytes) | CD19-FITC (4G7), CD14-PeCy7 (MoP9), CD56-APC (NCAM16.2), CD8-PE (SK1) and CD196-PE (11A9) from BD-Biosciences; CD161-FITC (191B8) from Miltenyi. |
| In vivo experiments:  Human and mouse CD45+ cells  Human CD3+CD4+  Human FoxP3+ Treg | Anti–human CD45-Brilliant Violet510 (HI30), CD4 Pacific Blue (RPA-T4), FoxP3 Alexa Fluor 488 (259D) from Biolegend. Anti-human CD3-APC-Vio770(BW264/56) and anti-mouse CD45 Percp from Miltenyi Biotech. CD25 APC (2A3) from BD-Biosciences |

**Figure S1**

**
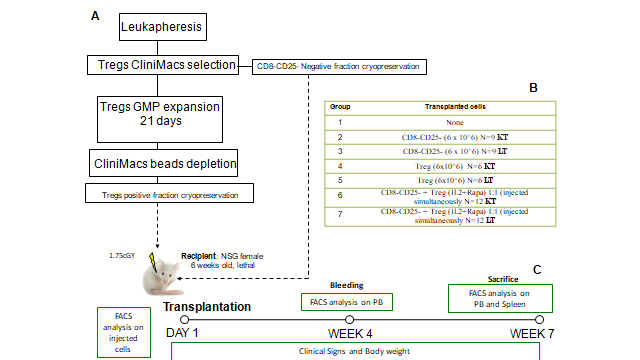
**

**Figure S2**

**
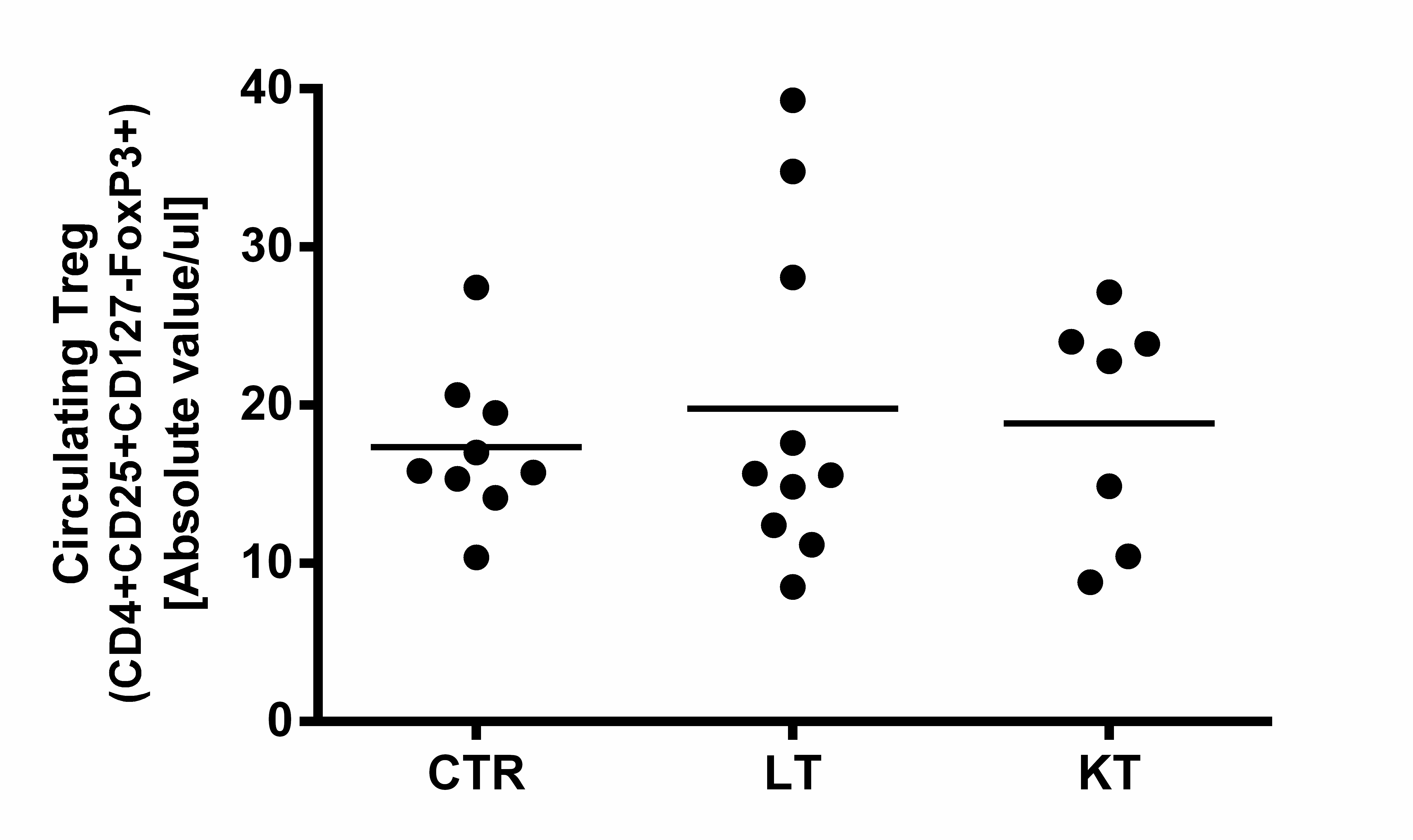
**
